# The effect of resveratrol and its methylthio-derivatives on EGFR and Stat3 activation in human HaCaT and A431 cells

**DOI:** 10.1007/s11010-014-2157-5

**Published:** 2014-07-26

**Authors:** Michal Cichocki, Hanna Szaefer, Violetta Krajka-Kuźniak, Wanda Baer-Dubowska

**Affiliations:** Department of Pharmaceutical Biochemistry, Poznań University of Medical Sciences, ul. Święcickiego 4, 60-781 Poznań, Poland

**Keywords:** Resveratrol, Methylthiostilbenes, HaCaT, A431, EGFR, Stat3

## Abstract

Epidermal growth factor receptor (EGFR) interacting with Stat3 is considered to be an attractive therapeutic target. In the current study, we investigated the effect of resveratrol and its two 4′-methylthio-*trans*-stilbene derivatives (3-M-4′-MTS; S2) (3,5-DM-4″-MTS; S5) on EGFR and Stat3 activation in human immortalized HaCaT keratinocytes and epidermoid carcinoma A431 cells. In the HaCaT cells both derivatives, similarly as resveratrol, decreased the total level of the EGFR receptor. In the A431 cells, resveratrol in the higher dose significantly (*p* < 0.05) reduced Y1173 and Y1068 EGFR residue phosphorylation, while S2 affected only the phosphorylation of the Y1068 residue. In this cell line, resveratrol in both tested doses and the S2 derivative in the lower concentration significantly diminished Stat3 binding capacity to the DNA consensus site. The effect of the tested compounds on Stat3 activation in HaCaT cells was only slightly affected. These results indicate that methylthiostilbenes are not more potent modulators of the EGFR/Stat3 complex than resveratrol and that introducing an additional methoxy group makes them less effective.

## Introduction

Keratinocytes are not only primary sensors of stressful conditions, but also major players of the extremely complex response in the skin when conducting the orchestrated recruitment and functions of the immune cells, fibroblasts, and vascular cells which are involved in inflammatory responses and wound healing. The epidermal growth factor receptor (EGFR), located on the cellular membrane of the keratinocytes, is widely recognized as a key regulator of numerous essential processes underlying skin development, homeostasis, and repair [1]. EGFR is a 170-kDa glycoprotein that consists of an extracellular receptor domain, a trans-membrane region, and an intracellular domain with tyrosine kinase function [2]. EGFR is expressed through all layers of the human epidermis, with the strongest presence in the basal layer of the epidermal keratinocytes. Several lines of evidence indicate the existence of two modes of EGFR signaling. The traditional cytoplasmic EGFR route involves transduction of mitogenic signals through the activation of numerous signaling cascades, signal transducers, and activators of transcription (Stats). In the nuclear pathway, activated EGFR undergoes direct nuclear translocation, where it interacts with other transcription factors possessing DNA-binding activity, including (Stat3), which leads to up-regulation of distinct genes controlling cell proliferation and DNA repair [3]. Stat3 is a cytoplasmic protein that is activated in response to cytokines and growth factors and is considered as molecular machinery that regulates cell fate determination, renewal, differentiation, and apoptosis of various cell types, especially that ones at the embryonic developing stages [4, 5]. On activation, Stat3 molecules are translocated to nucleus, where they activate transcription of a series of target genes including c-Myc, survivin, and cyclin D1 that are closely associated with the growth, survival, and progression of cancer cells [5]. Indeed, in cancerous cells Stat3 constitutive activation is common, which is likely due to the aberrant activity of Stat3’s upstream signaling pathways, such as, EGFR, HER2, Src, and JAK2. Tumorigenic Stat3 activation has been frequently linked to more malignant cancer behaviors, including growth, epithelial-mesenchymal transition, migration, invasion, and metastasis. Stat3 activation is also associated with tumor survival and therapeutic resistance [6]. Many studies also have confirmed, the importance of Stat3 in skin carcinogenesis, skin cancer prevention, and treatment [4].

There is steadily growing interest in skin protection by plant polyphenols, although the mechanisms by which these natural compounds exert their beneficial effects are not fully understood [7]. One such polyphenol is resveratrol (3,5,4′-trihydroxy-*trans*-stilbene), a naturally occurring phytoalexin and the most extensively studied stilbene derivative. This compound has been shown to exert several beneficial effects, including cancer chemopreventive activity, in several preclinical studies [8, 9].

However, an important issue connected with the future application of resveratrol in disease management is its low bioavailability due to its rapid metabolism in mammals [10]. A strategy targeted at discovering and defining novel analogs of resveratrol has been assumed. These analogs should have the same structural backbone of resveratrol, with chemical modifications resulting in superior efficacy [11].

Our earlier studies showed that introduction of the methylthio-group into the stilbene core may influence the efficacy and selectivity of the inhibitory potency of these compounds toward the P450 isozymes, CYP1A1 and 1B1 [12, 13]. Other authors showed that substitution of the 4′ oxygen atom with a less electronegative sulfur atom also reduced toxicity toward the HEK 293 cells and enhanced the compound’s ability to activate human SIRT1 [14].

The aim of the present study was to further explore the activity of resveratrol and its methylthio-derivatives by evaluating their effect on EGFR and Stat3 activation. Two 4′-methylthio-*trans*-stilbene derivatives possessing one (3-M-4′-MTS; S2) and two (3,5-DM-4′-MTS; S5) methoxy groups were assessed, and immortalized human HaCaT keratinocytes and human epidermoid carcinoma A431 cells differing in EGFR constitutive expression were applied as an experimental model.

## Materials and methods

### Chemicals

Resveratrol (purity 99 %), dithiothreitol, antibiotic solution (10^4^ U penicillin, 10 mg streptomycin, 25 μg amphotericin B), bovine serum albumin (BSA), dimethyl sulfoxide (DMSO), fetal bovine serum (FBS), Dulbecco’s Modified Eagle’s Medium (DMEM), and Tris were purchased from Sigma Chemicals (St. Louis, MO, USA). Primary and secondary antibodies against EGFR, Y1173-EGFR, Y1068-EGFR, Stat3, c-Myc, Bcl-xL, and β-actin were supplied by Santa Cruz Biotechnology (Santa Cruz, CA, USA). SDS-PAGE Gels (7.5 %, 10 %, 12 %) and the Western blotting detection system were purchased from Bio-Rad Laboratories (Hercules, CA, USA). All other compounds were readily available commercial products.

**Fig. 1:**
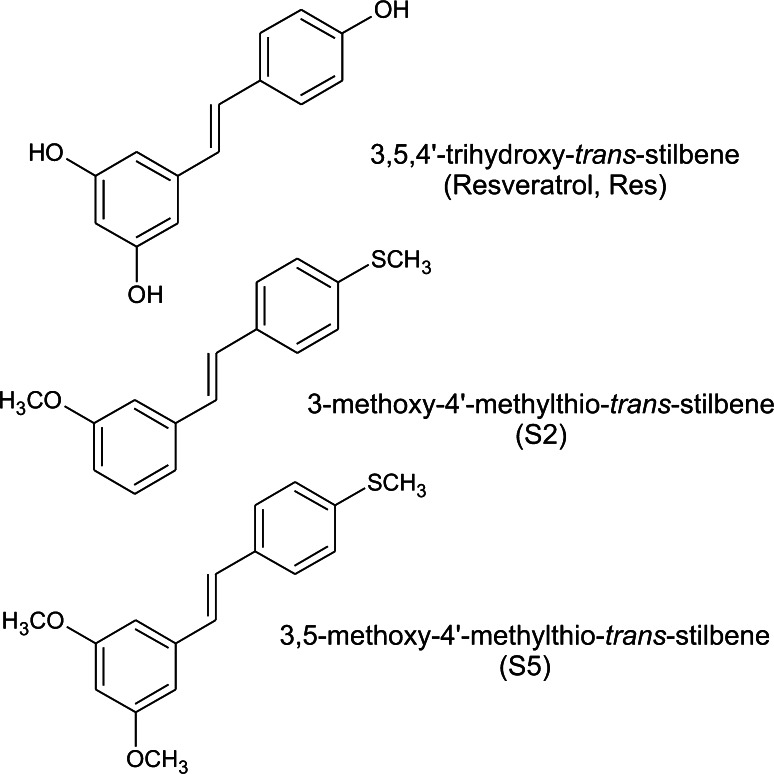
Structures of resveratrol and methylthiostilbenes

3-methoxy-4′-methylthio-*trans*-stilbene (S2) and 3,5-dimethoxy-4′-methylthio-*trans*-stilbene (S5) were synthesized as described previously [12]. Their structures are shown in Fig. 1.

### Cell culture and treatment

Spontaneously immortalized human keratinocyte HaCaT cells, purchased from Cell Lines Service (CLS, Germany), and human epidermoid carcinoma A431 cells, obtained from Deutsche Sammlung von Mikroorganismen und Zellkulturen (DSMZ, Germany), were grown in DMEM containing 10 % FBS supplemented with antibiotics (penicillin, streptomycin, and amphotericin B). The cells were incubated at 37 °C in an atmosphere consisting of 95 % air and 5 % CO_2_ in a humidified incubator until they reached 80 % confluency. 1 × 10^6^ cells were seeded in 100 mm ø culture dishes. After 24 h of preincubation in DMEM containing 5 % of FBS, the cells were treated with either 20 and 60 μM resveratrol, 20 and 60 μM S2 derivative, 5 and 20 μM S5 compound, or 0.1 % DMSO (control cells). Incubation was continued for a subsequent 24 h, then the cells were harvested. The preliminary experiments were performed in which the effect of resveratrol and its derivatives on EGFR at different time points (2, 4, 12, and 24 h) was estimated. No significant differences in time-course response were observed. For this reason, only one time point of 24 h was chosen for further studies.

### Cell viability assay

The effects of resveratrol and methylthiostilbenes on cell viability were assessed with MTT assay according to standard protocols. Since the data on cytotoxicity of these compounds in HaCaT cells were presented previously [15], the MTT test was performed only in A431 cells. Briefly, 10^4^ cells were seeded per well in a 96-well plate. After 24 h of preincubation in DMEM containing 5 % FBS, the tested compounds were added to the culture medium in various concentrations (0–200 μM), and the cells were incubated for a subsequent 72 h. The DMSO concentration did not exceed 0.1 %. After 72 h, the cells were washed twice with PBS buffer, and a fresh medium containing MTT salt (0.5 mg/ml) was added. After 4 h of incubation, formazan crystals were dissolved in acidic isopropanol and absorbance was measured at 540 and 690 nm. All of the experiments were repeated three times, with at least three measurements per assay.

In all of the subsequent experiments, non-toxic concentrations of methylthiostilbenes and resveratrol (viability level above 70 %) were used ranging from 5 to 60 µM, depending on compound.

### Cell fractionation

Cytosol and nuclear extracts from HaCaT and A431 cells were prepared using the Nuclear/Cytosol Fractionation Kit (Active Motif, Carlsbad CA, USA). Whole cell lysates were prepared from HaCaT and A431 cells using the standard RIPA buffer.

### Western blot analysis

Immunoblot assay was used to determine the level of EGFR, Y-1173-EGFR, Y-1068-EGFR Stat3, c-Myc, and Bcl-xL proteins. The protein content in the samples was determined with the Lowry method. Whole cell lysates (EGFR, Y-1173-EGFR, Y-1068-EGFR, c-Myc, Bcl-xL), nuclear and cytosolic fraction (Stat3) (50–100 μg) were separated on 10 % or 7.5 % or 12 % SDS-PAGE gels, and the proteins were transferred to nitrocellulose membranes [16, 17]. After blocking with 10 % skimmed milk, the proteins were probed with rabbit polyclonal EGFR, rabbit polyclonal Y-1173-EGFR, goat polyclonal Y-1068-EGFR, rabbit polyclonal Stat3, mouse monoclonal c-Myc, mouse monoclonal Bcl-xL, and rabbit polyclonal β-actin antibodies. The β-actin protein was used as an internal control. Alkaline phosphatase-labeled anti-rabbit IgG, anti-mouse IgG, or anti-goat IgG were used as the secondary antibodies in the staining reaction. The amount of immunoreactive product in each lane was determined using Quantity One software (BioRad Laboratories, Hercules, CA, USA). Values were calculated as relative absorbance units (RQ) per mg of protein.

### Stat3—DNA-binding assay

Stat3 activation was assessed by estimating Stat3—DNA-binding capacity assay by the means of enzymatic immunoassay according to Jin et al. [18] using commercial kits (TransAM Stat Family; Active Motif, Carlsbad CA, USA) and following the manufacturer’s instructions. The amounts of Stat3 protein contained in DNA-binding complex were measured, and the Stat consensus binding site (5′-TTCCCGGAA-3′) was immobilized on ELISA microplates as a bait. The results were expressed as absorbance (OD_450nm_) per mg of protein.

### Statistical analysis

The statistical analysis was performed by one-way ANOVA. The statistical significance between the experimental groups and their respective controls was assessed by Tukey’s post hoc test, with *p* < 0.05 being considered significant.

## Results

### The effect of resveratrol and methylthiostilbenes on cell viability in A431 cells

**Fig. 2:**
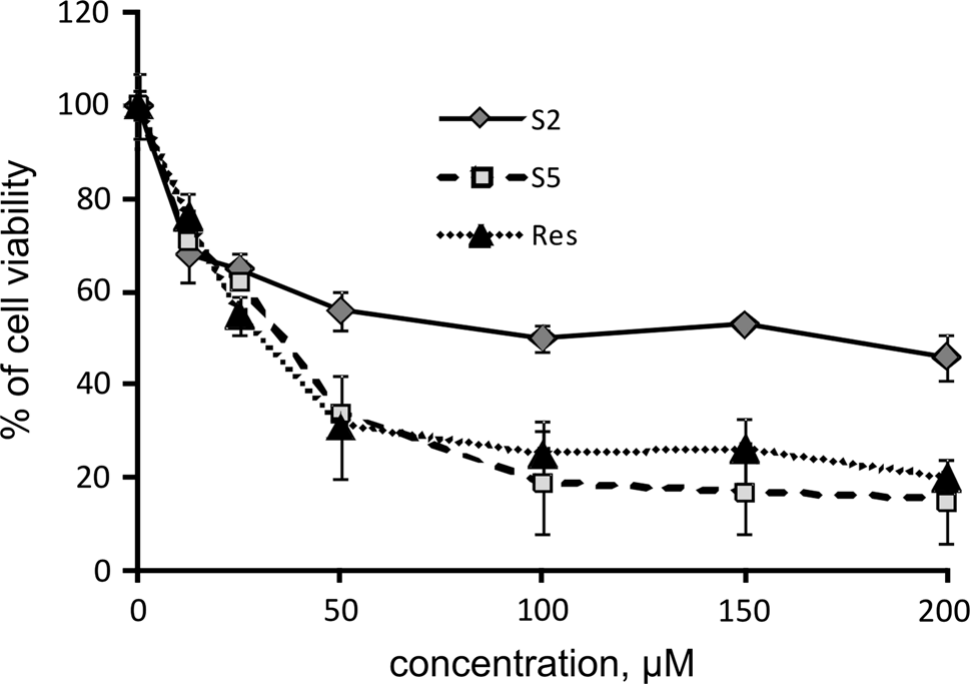
Effect of resveratrol (RES) and methylthiostilbenes (S2, S5) on the viability of A431 cells. The mean values ± SEM from three independent experiments run in triplicate are shown. The viability of vehicle-treated cells was considered 100 %

Figure 2 presents data on the effect of stilbenes on A431 viability. Within the concentration range of 2–200 μM, the tested compounds reduced the viability of the A431 cells in a dose-dependent manner. In this cell line, cytotoxicity of S5 was similar to the effect of resveratrol and compound S2 was less toxic. However, compound S2 exhibited higher toxicity toward A431 cells in comparison with HaCaT cells (IC_50_ 182.66 ± 38.71 vs 141.28 ± 2.80 µM). Resveratrol also displayed higher toxicity toward A431 cells than to HaCaT cells (IC_50_ 85.23 ± 29.17 vs 49.44 ± 3.76 µM), while the toxicity of S5 was similar in both cell lines [15].

### The effect of resveratrol and methylthiostilbenes on EGFR activation in HaCaT and A431 cells

**Fig. 3:**
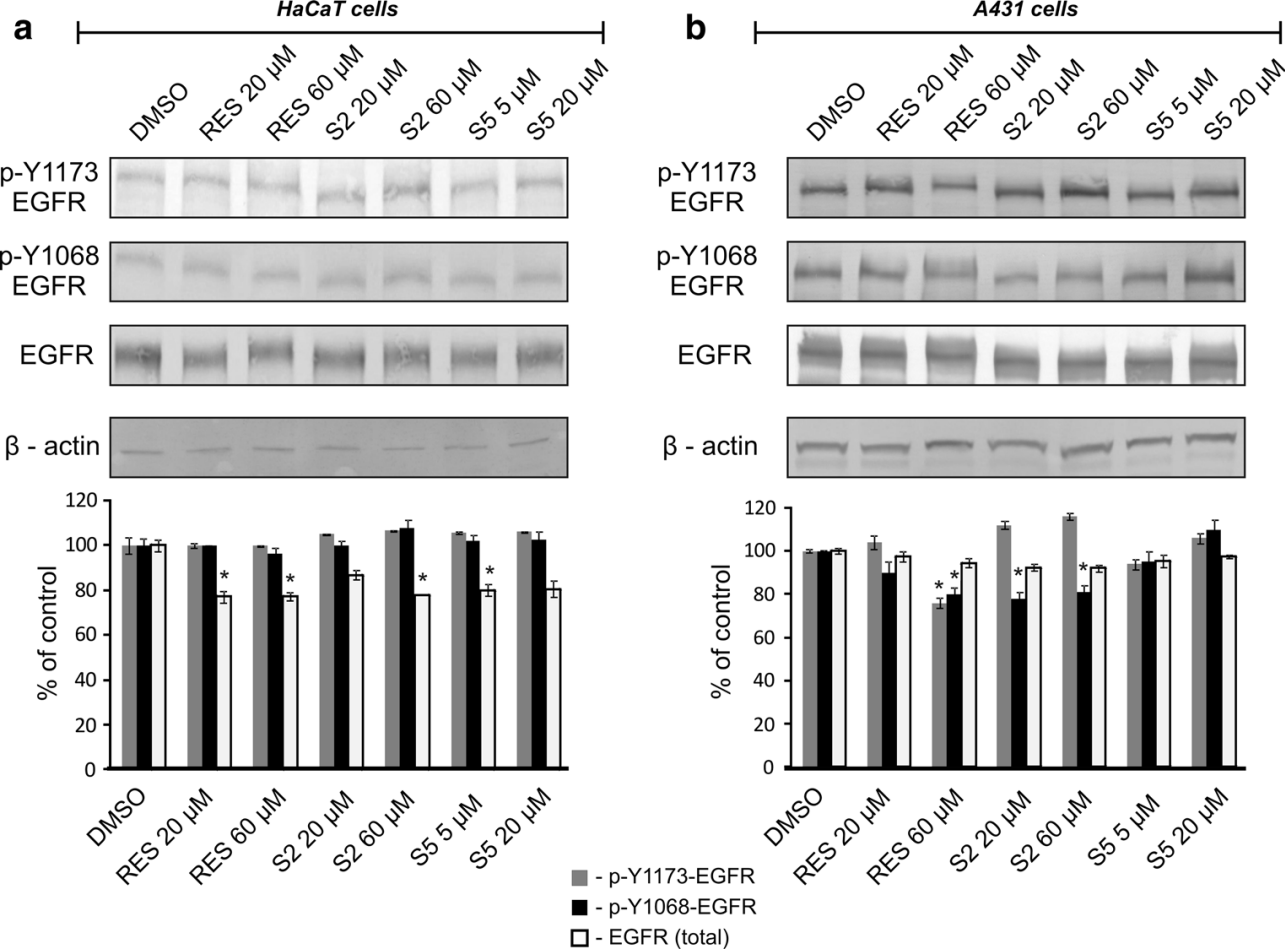
Effect of resveratrol and methylthiostilbenes on EGFR activation in HaCaT (**a**) and A431 (**b**) cells. Levels of the protein were assessed by Western blot analysis. Representative immunoblots and data (mean ± SEM) from three independent experiments run in duplicate are presented. β-actin was used as an internal control. The *asterisk* above the *bar* denotes statistically significant differences from the control cells (DMSO), *p* < 0.05

The effects of resveratrol and methylthiostilbenes on both the EGFR protein level and activation are shown in Fig. 3. In the HaCaT cells both derivatives, similarly to resveratrol, decreased the total level of the EGFR protein but did not affect the level of the phosphorylated Y1173 or Y1068 forms of this receptor (Fig. 3a). A different effect was observed in the A431 cells (Fig. 3b), where none of the compounds affected the total level of the EGFR protein, but the level of EGFR phosphorylation of the Y1068 residue was significantly (*p* < 0.05) reduced by resveratrol in the higher dose and by S2 in both doses. Additionally, resveratrol in the higher dose (60 µM) significantly diminished the level of Y1173 phosphorylation.

### The effect of resveratrol and methylthiostilbenes on Stat3 activation in HaCaT and A431 cells

**Fig. 4:**
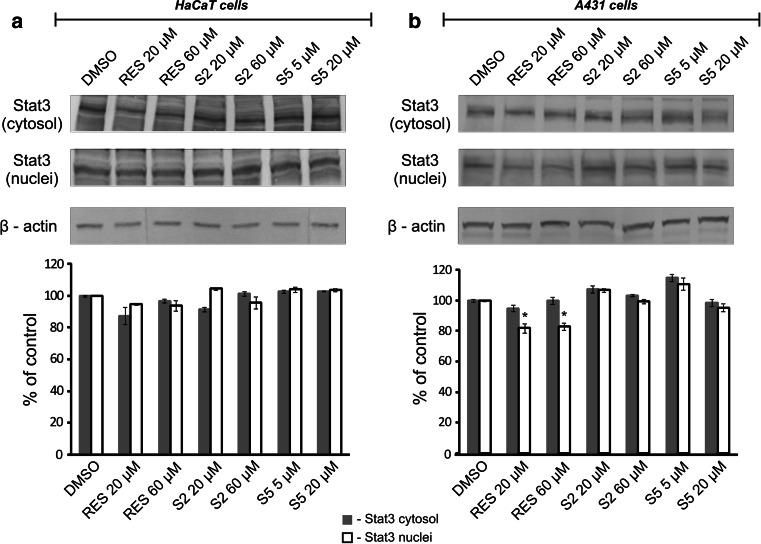
Effect of resveratrol and methylthiostilbenes on Stat3 translocation in HaCaT (**a**) and A431 (**b**) cells assayed by Western blot analysis. Representative immunoblots and data (mean ± SEM) from three independent experiments run in duplicate are presented. β-actin was used as an internal control. The *asterisk* above the *bar* denotes statistically significant differences from the control cells (DMSO), *p* < 0.05

**Fig. 5:**
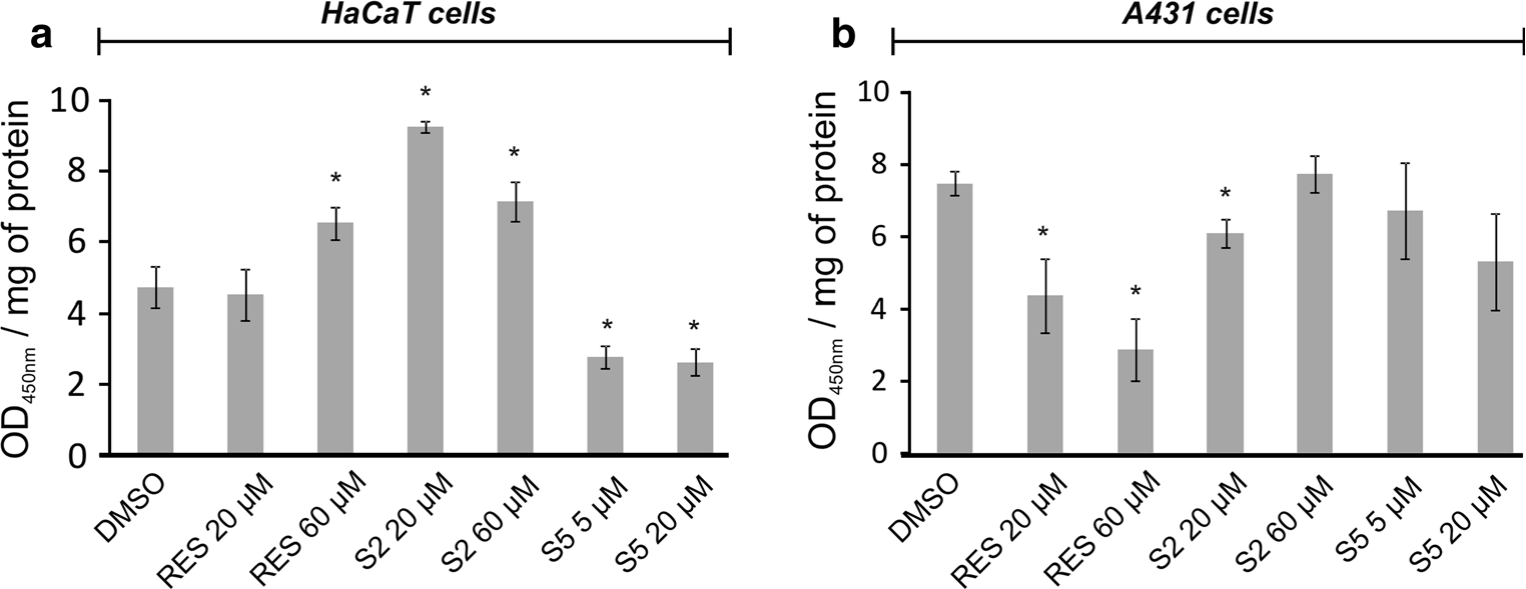
Effect of resveratrol and methylthiostilbenes on Stat3 activation in HaCaT (**a**) and A431 (**b**) cells. Activated Stat3 was assessed in terms of the amount of Stat3 contained in the DNA-binding complexes extracted from the nuclei that were isolated from the cells. The Stat consensus site (5′-TTCCCGGAA-3′) was immobilized on ELISA plates as bait. *Bars* represent mean ± SEM from three independent experiments run in duplicate. The *asterisk* above the *bar* denotes statistically significant differences from the control cells (DMSO), *p* < 0.05

Figures 4 and 5 show the effect of resveratrol and methylthiostilbenes on Stat3 activation, estimated in terms of translocation of the Stat3 protein from the cytosol to the nuclear fraction (Fig. 4), and measured by Western blot and the binding capacity to DNA consensus sequences (Fig. 5). None of the tested compounds changed the level of Stat3 in the cytosolic or nuclear fractions of the HaCaT cells (Fig. 4a). However, resveratrol in the higher dose, and S2 in both doses, significantly increased Stat3 binding capacity to the DNA consensus site (Fig. 5a) in these cells. The S5 derivative had just the opposite effect, as in both of the tested doses this compound significantly diminished Stat3-DNA binding by approximately 50 %.

In human epidermoid carcinoma A431 cells, resveratrol was the only compound affecting the total level of the Stat3 protein, as it significantly diminished this transcription factor content in the nuclear fraction by 20 % (Fig. 4b). Also, resveratrol significantly diminished the Stat3 binding capacity to the Stat consensus sequence in a dose-dependent manner (Fig. 5b), while S2 was active to a much lesser extent and only in the lower concentration. In the case of S5, which significantly affected Stat3 binding in the HaCaT cells, no statistically significant effect was observed in human skin carcinoma A431 cells.

### The effect of resveratrol and methylthiostilbenes on Stat3 downstream targeted genes in HaCaT and A431 cells

**Fig. 6:**
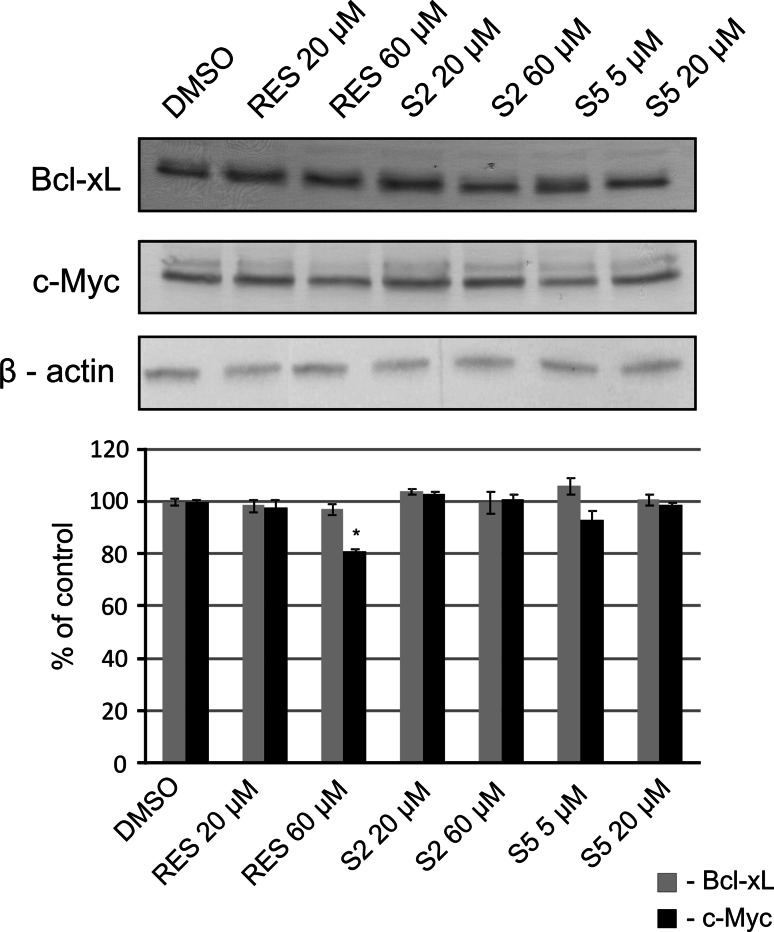
Effect of resveratrol and methylthiostilbenes on Stat3 downstream targeted genes in A431 cells assayed by Western blot analysis. Representative immunoblots and data (mean ± SEM) from three independent experiments run in duplicate are presented. β-actin was used as an internal control. The *asterisk* above the *bar* denotes statistically significant differences from the control cells (DMSO), *p* < 0.05

Figure 6 shows the effect of resveratrol and methylthiostilbenes on c-Myc and Bcl-xL protein level in A431 cells. In these cells, only resveratrol in the higher dose (60 µM) significantly diminished the level of c-Myc. The level of these proteins in HaCaT cells was too low for quantitative statistical evaluation.

## Discussion

Overexpression and/or increased activity of EGFR are the key characteristics of human tumors and are frequently linked to more aggressive tumor behavior, including increased proliferation, metastasis, and therapeutic resistance [19].

Several reports described the nuclear localization of EGFR which resulted from translocation directly into the nucleus and which omitted the traditional pathway that requires the activation of signaling cascades [20, 21]. Although EGFR possesses transactivational activity, it lacks a DNA-binding domain and requires a DNA-binding transcription cofactor for its transcriptional function. In this regard, it was shown that EGFR physically and functionally interacts with Stat3 in the nucleus, leading to transcriptional activation of the inducible nitric oxide synthase (iNOS) gene. Thus, combining agents targeting both EGRF and Stat3 may improve the chemopreventive and/or therapeutic effect. Indeed, the combination of AG490 and AG1478 was more potent than the inhibitor alone in killing EGFR-overexpressing cells, i.e., A431 and MDA-MB-468 (human breast carcinoma) [22]. Single agents affecting both of these targets are equally desired.

Resveratrol has been demonstrated to affect a multitude of signal transduction pathways associated with tumorigenesis [23]. In mouse skin, reduction of the iNOS protein level and activity elevated by the tumor promoter, TPA, were observed as a result of pretreatment with resveratrol, and even more by its naturally occurring analog, pterostilbene.

This observation may imply the involvement of altered EGFR/Stat3 activation in TPA induction of iNOS in mouse skin. Moreover, it suggests that resveratrol analogs might be more efficient in the modulation of this pathway.

In this study, we compared the effect of resveratrol and its methylthio-derivatives on the expression and activation of EGFR and Stat3 in two cell lines which differed in EGFR expression. While the A431 cells were characterized by EGFR-overexpression, spontaneously immortalized keratinocytes, HaCaT cells, have low constitutive EGFR activity. Moreover, EGFR activation in HaCaT cells had no effect on Stat3 Y705 phosphorylation [24] .

Activation of EGFR results in autophosphorylation of the C-terminal region. The major sites of phosphorylation are the Y1068 and Y1173 tyrosine residues [25].

Treatment of the HaCaT cells with either resveratrol or methylthiostilbenes did not affect the phosphorylation of these residues, although the total level of the EGFR protein was significantly reduced. This may suggest that stilbenes might reduce the constitutive expression of EGFR or stimulate its degradation. The latter may occur e.g., through activating of ubiquitination and sorting machinery such as ESCRT [26]. It also cannot be excluded that other EGFR residues which were not evaluated in this study might be affected and contributed to overall EGFR protein reduction. The possible candidates are 1045 and 1173 tyrosine residues which were found to be phosphorylated in A431 cells, but were not evaluated yet in HaCaT cells [27].

In EGFR-overexpressing A431 cells, a significant reduction of EGFR with both phosphorylated residues was observed as a result of resveratrol at higher dose treatment. Compound S2, 3-methoxy-4′-methylthio-*trans*-stilbene, significantly reduced the level of EGFR with the phosphorylated Y1068 tyrosine residues. Introducing an additional methoxy group to the stilbene ring made this compound ineffective as an EGFR activation modulator.

Reducing the phosphorylation of the Y1068 residue by resveratrol and compound S2 may have further consequences, as we found in our previous studies that phosphorylation of this tyrosine correlated with AP-1 activation in mouse skin. This transcription factor is involved in COX-2 and iNOS induction, which is reduced by resveratrol in this model [28, 29].

Reduced activation of EGFR by resveratrol and its S2 analog was connected with reduced activation of Stat3. Resveratrol treatment reduced the binding level of this transcription factor to its consensus oligonucleotide measured in terms of its amount in the DNA-binding complex extracted from the nuclear fraction of the A431 cells. Thus this observation indirectly confirmed the interaction of EGFR and Stat3 in these cells, as was described by Lo et al. [30].

On the other hand, in the HaCaT cells the S5 stilbenoid reduced the binding of Stat3 to its consensus sequence, while resveratrol and the S2 compound increased Stat3 activation. The above-mentioned studies by Quadros et al. [24] demonstrated, however, that DNA binding of activated Stat3 is restricted to malignant cells (SCC) and strictly dependent on EGFR activation. Thus the results of our current experiments may suggest that activation of Stat3 in nonmalignant cells, such as HaCaT, in response to stilbene treatment occurs in an EGFR-independent way. Further studies are required to confirm this suggestion. Collectively, the results of our present study indicate that stilbenoids may affect both EGFR and Stat3 in malignant, EGFR-overexpressing tumor cells.

However, modification of the stilbene ring does not affect the EGFR/Stat3 system to a greater extent than resveratrol.

## Abbreviations

A431: Human epidermoid carcinoma cells
DMEM: Dulbecco’s Modified Eagle’s Medium
DMSO: Dimethyl sulfoxide
EGFR: Epidermal growth factor receptor
FBS: Fetal bovine serum
HaCaT: Spontaneously immortalized human keratinocytes
MAPKs: Mitogen-activated protein kinases
Res: Resveratrol
S2: 3-Methoxy-4′-methylthio-*trans*-stilbene (3-M-4′-MTS)
S5: 3,5-Dimethoxy-4′-methylthio-*trans*-stilbene (3,5-DM-4″-MTS)
Stat: Signal transducer and activator of transcription
